# One-Step Low Temperature Synthesis of CeO_2_ Nanoparticles Stabilized by Carboxymethylcellulose

**DOI:** 10.3390/polym15061437

**Published:** 2023-03-14

**Authors:** Vasily V. Spiridonov, Andrey V. Sybachin, Vladislava A. Pigareva, Mikhail I. Afanasov, Sharifjon A. Musoev, Alexander V. Knotko, Sergey B. Zezin

**Affiliations:** 1 Department of Chemistry, Lomonosov Moscow State University, Leninskie Gory 1-3, 119991 Moscow, Russia; sybatchin@mail.ru (A.V.S.);; 2 Faculty of Materials Science, Lomonosov Moscow State University, Leninskie Gory 1-73, 119991 Moscow, Russia

**Keywords:** carboxymethyl cellulose, cerium oxide, nanocomposite, cerium nanoparticles, stabilization, microscopy

## Abstract

An elegant method of one-pot reaction at room temperature for the synthesis of nanocomposites consisting of cerium containing nanoparticles stabilized by carboxymethyl cellulose (CMC) macromolecules was introduced. The characterization of the nanocomposites was carried out with a combination of microscopy, XRD, and IR spectroscopy analysis. The type of crystal structure of inorganic nanoparticles corresponding to CeO_2_ was determined and the mechanism of nanoparticle formation was suggested. It was demonstrated that the size and shape of the nanoparticles in the resulting nanocomposites does not depend on the ratio of the initial reagents. Spherical particles with a mean diameter 2–3 nm of were obtained in different reaction mixtures with a mass fraction of cerium from 6.4 to 14.1%. The scheme of the dual stabilization of CeO_2_ nanoparticles with carboxylate and hydroxyl groups of CMC was proposed. These findings demonstrate that the suggested easily reproducible technique is promising for the large-scale development of nanoceria-containing materials.

## 1. Introduction

Cerium oxide nanoparticles (nanoceria) are of interest in a wide range of applications, especially in the field of biomedicine. Many chemical methods for the synthesis of nanoceria are reported in the literature. Precipitation methods such as co-precipitation, chemical precipitation, microwave, sonochemical, hydrothermal, reverse-co-precipitation, and microwave–hydrothermal methods are basic procedures of synthesis [1,2,3,4,5,6,7,8,9,10,11,12,13,14]. The key disadvantage of these methods for obtaining cerium-containing nanoparticles is the requirement to conduct the reactions at high temperatures. That, in turn, leads to the formation of particles of arbitrary sizes and shapes. As a result, no control of the geometric and morphological parameters of nanoparticles can be achieved. Moreover, the problem of stabilization of cerium-containing nanoparticles from aggregation and precipitation also remains relevant. The most commonly used approach to stabilize cerium oxide nanoparticles is modification of their surfaces with surfactants [15]. A significant drawback of this approach is the impossibility of complete purification of the formed nanocomposites from surfactants and, as a result, limitation of the use of such materials for biomedical purposes. Eco-friendly examples of green synthesis of nanoceria were reported as well, for example, the synthesis of nanoceria using nutrient *Salvia macrosiphon Boiss* seeds [16]. However, such approaches did not find wide distribution.

A high degree of biocompatibility, low toxicity, and catalytic activity of nanodispersed cerium dioxide make it possible to consider it as a promising material for biomedical applications as antioxidant, anticancer, and antibacterial agents [17,18,19]. That is why cerium oxide nanoparticles, which have biomimetic and antioxidant activity, are now increasingly being used in medicine.

The influence of nanocrystalline cerium dioxide in the protection of living cells from oxidative stress is of particular interest. The uniqueness of cerium dioxide nanoparticles is due to the fact that they can exist in different oxidation states, Ce^3+^ and Ce^4+^, which distinguishes them from most other rare earth metals, which exist predominantly in the trivalent state [20,21]. The biological activity of cerium dioxide nanoparticles is determined by oxygen nonstoichiometry, which depends on the size of the nanoparticle and the nature of the surface ligand. It has been shown that cerium-containing nanoparticles can act as superoxide dismutase, catalases, oxidases, and oxidoreductases [22]. In this case, the efficiency of radical neutralization is proportional to the concentration of Ce^3+^ ions on the surface of nanoparticles. In addition, the size and state of the surface of CeO_2_ particles determine the possibility of inactivation of superoxide free radicals, preventing oxidative stress in cells [22]. The most effective anti-oxidative-stress activity cerium oxide nanoparticles demonstrate has been shown in alkaline and neutral (physiological) media [23,24].

Along with the complicated preparation procedures of cerium-containing nanoparticles, there exists the important problem of their stabilization against aggregation (agglomeration). Different approaches to the stabilization of nanoparticles, such as gold, silver, and iron oxides as well as cerium oxide, are widely discussed in the literature. For example, various stabilizers have been found to be effective in preventing the agglomeration of nanosized particles, including thiols, carboxylic acids, surfactants, and polymers [25,26,27,28,29,30]. In general, some stabilizers are not environmentally friendly, while others are prohibitively expensive. The key requirements for the stabilizers for the nanocomposites proposed for biomedical applications are the following: the stabilizer must be capable of specific interactions with nanoparticles to inhibit their growth and they should be harmless to living organisms and the environment. As well, it is necessary to use a stabilizer that provides a longer effective stabilization, which will facilitate the shelf-life of nanoparticles.

In this work, for the preparation of stable cerium oxide (CeO_2_) nanoparticles, sodium salt of carboxymethyl cellulose was used. CMC is water soluble and commonly used in the food industry [30]. In addition, CMC has been successfully used as an effective stabilizer in the preparation processes of superparamagnetic iron oxide nanoparticles and Ag nanoparticles [31,32]. CMC is environmental friendly and harmless to living organisms. It is extremely important that CMC is a polyelectrolyte and contains carboxylate groups along with hydroxyl groups in monomer units [33]. Polyelectrolytes are promising components for the effective stabilization of cerium-containing nanoparticles. The efficiency of preventing aggregation can be achieved due to the ability of charged groups in the main chain to interact electrostatically with the surface of nanoparticles. In addition, the natural origin of CMC stabilizer will make it possible to obtain biocompatible nanocomposite materials.

In this communication, we propose a facile original method for the preparation of fine cerium oxide nanoparticles, which was carried out by the reduction of the Ce^4+^ complex salt with sodium borohydride in the presence of CMC at room temperature under aerobic conditions. The suggested technique allows one to easily separate the resulting product from the non-reacted initial compounds and side products with dialysis. The obtained nanocomposite could be stored as a colloid stable solution or as a lyophilized powder without losing its quality.

## 2. Materials and Methods

### 2.1. Materials

The following reagents were used in this work: sodium salt of CMC with molecular mass 90.000 Da DS 0.7 (Merck, Rahway, NJ, USA, Analytical grade), ammonium cerium nitrate, (NH_4_)_2_Ce(NO_3_)_6_ (Reakhim, Russia, Special Purification grade), and sodium borohydride (99%, Pulver, Belgium, Special Purification grade).

### 2.2. Nanoparticle Synthesis Procedure

The synthesis of composite materials based on the sodium salt of carboxymethyl cellulose and cerium oxide nanoparticles was carried out according to the following procedure. To 2.5 mL of a 2 wt.% solution of CMC, 5 mL of the solution of (NH_4_)_2_Ce(NO_3_)_6_ was added dropwise under vigorous stirring. The masses of (NH_4_)_2_Ce(NO_3_)_6_ in solutions were varied from 6.3 to 17.6 mg. Then, 0.5 mL solution containing 3 mg to 5 mg of NaBH_4_, respectively, was added to the reaction mixture under intensive stirring. The resulting volume of the reaction mixture was 8 mL. The obtained solution was stirred for 12 h. Then, purification from low-molecular-weight components was carried out using dialysis (in dialysis bags, Sigma, MWCO~12 kDa) against water for 24 h. The resulting products were lyophilized.

### 2.3. Research Methods for Cerium-Containing Nanocomposites

#### 2.3.1. UV Spectroscopy

The determination of the cerium content in the composites was carried out using the UV spectroscopy method. The measurements were carried out on a Specord M40 device from Carl Zeiss (Jena, Germany) in the spectral range from 280 to 500 nm [34,35]. Sample solutions were prepared to record the UV spectra. A calibration graph was built according to the method given in [15]. To construct this calibration graph, weighed amounts of cerium ammonium nitrate (0.5 mg, 1 mg, 1.5 mg, and 2 mg) were dissolved in 100 µL of concentrated H_2_SO_4_. Then, 10 mL of an aqueous solution containing 0.1% wt. of silver nitrate and 0.2 g of ammonium persulfate was added to the solutions. After that, the UV spectra of the obtained solutions were recorded in the wavelength range from 200 to 500 nm and the absorption intensity was measured at a wavelength of 310 nm (D). Absorption spectra of the solutions containing cerium ions of various concentrations and a calibration curve are presented in Supplementary Materials Figure S1.

#### 2.3.2. Transmission Electron Microscopy (TEM)

Structural studies were carried out with transmission (tunneling) electron microscopy (TEM) on a JEM-100B setup (JEML) equipped with an attachment for X-ray phase analysis [35]. The samples were prepared by applying a drop of an aqueous solution containing the test substance to a copper grid with further drying in an air atmosphere. The samples were examined without preliminary contrasting. The series of images was analyzed using ImageJ software. The obtained results were used to calculate the sizes of the nanoparticles fractions and their differential share.

#### 2.3.3. X-ray Diffraction Analysis (XRD)

X-ray studies were carried out on a Rigaku D/Max2500 diffractometer with a rotating anode (Japan). The survey was carried out in reflection mode (Bragg–Brentano geometry) using CuKα1 radiation (cf. wavelength λ = 1.54183 Å). The generator operation parameters were: accelerating voltage 40 kV, tube current 200 mA. The survey was carried out in quartz cuvettes without averaging rotation. Solvents were not used to fix the powder samples. The recording parameters were: angle interval 2θ = 2°–60°, step (in 2θ) 0.02°, spectrum recording rate 5°/min [34]. Silicon powder was used as an internal standard for correction.

Qualitative analysis of the obtained radiographs was performed using the WinXPOW software package (version 1.06) using the ICDDPDF-2 database.

#### 2.3.4. IR Spectroscopy

The nature of the interaction between the samples was studied using IR spectroscopy on a Specord M80 instrument (Carl Zeiss, Jena, Germany). Studies were carried out in the absorption mode by preparing tablets from KBr (matrix) [35].

#### 2.3.5. Raster (Scanning) Electron Microscopy (SEM)

The microstructure of the samples was studied using a scanning electron microscope with a LEO SUPRA 50VP field emission source (Carl Zeiss, Jena, Germany). For the study, the samples were glued on a metal table using a conductive carbon adhesive tape and a layer of carbon or chromium was deposited on them (sputtering unit Quorum 150T—(UK), voltage 1000–1200 V, current strength 5–10 mA, deposition time 5–10 min [34]). The accelerating voltage of the electron gun was 5–20 kV. Images were obtained in secondary electrons (detector SE2) at magnifications up to 100,000× and recorded in digitized form on a computer.

## 3. Results

An original technique was used for the first time to obtain CMC–CeO_2_ nanocomposites. The CMC–CeO_2_ nanocomposites were obtained by treating CMC with solutions of (NH_4_)_2_Ce(NO_3_)_6_ and a reducing agent (NaBH_4_) at room temperature. A feature of the proposed approach is the formation of cerium oxide in an aqueous medium simultaneously in the presence of a strong reducing agent and atmospheric oxygen. The need to use a reducing agent is due to the chemical specificity of this process, which, apparently, includes the following stages [36,37]:

Ce^4+^ + BH_4_^−^ → Ce^3+^ + B(OH)_3_ + H_2_ (1)

NaBH_4_ + 4H_2_O → NaB(OH)_4_ + 4H_2_ (2)

NaB(OH)_4_ → NaOH + B(OH)_3_ (3)

Ce^3+^ + 3OH^−^ → Ce(OH)_3_ (4)

4Ce(OH)_3_ + O_2_ → 4CeO_2_ + 6H_2_O (5)

During the synthesis, the concentration of CMC was kept constant, while the concentration of the cerium salt, the source of cerium (IV) ions, changed six-fold (see details in Table 1). This made it possible to trace the effect of the molar ratio of components in the reaction system on the composition of the final product.

**Figure 1:**
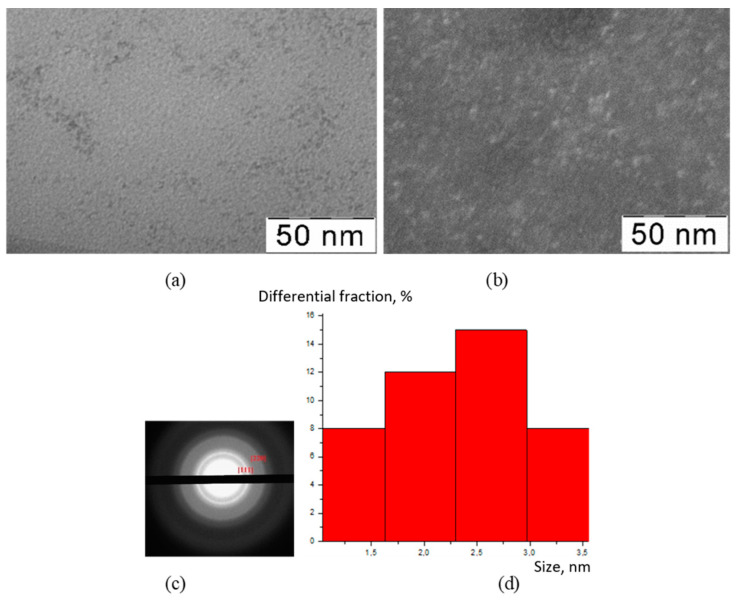
TEM image (**a**), TEM image in dark field (**b**), diffractogram (**c**), nanoparticle size distribution (**d**) of nanoceria-containing composites of CMC with 7.1 wt.% Ce^4+^.

**Figure 2:**
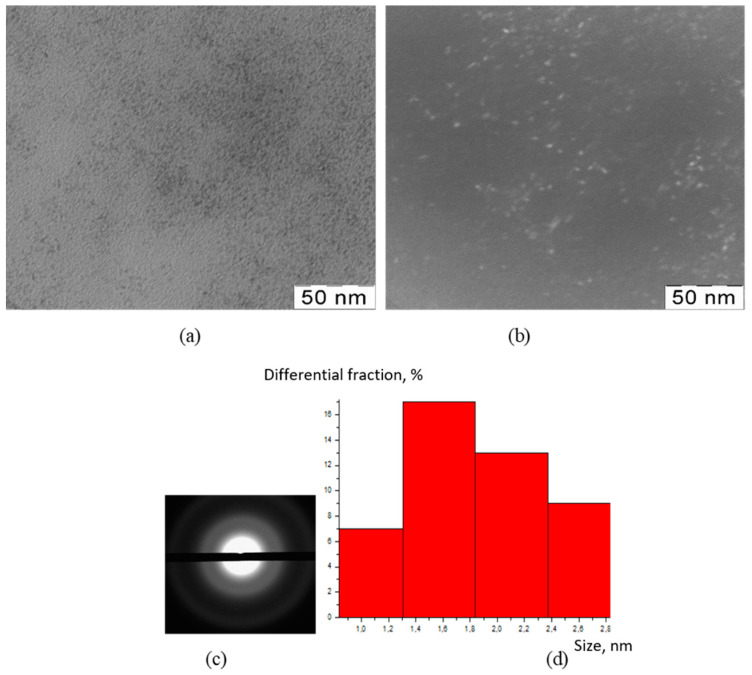
TEM image (**a**), TEM image in dark field (**b**), diffractogram (**c**), nanoparticle size distribution (**d**) of nanoceria-containing composites of CMC with 9.0 wt.% Ce^4+^.

**Figure 3:**
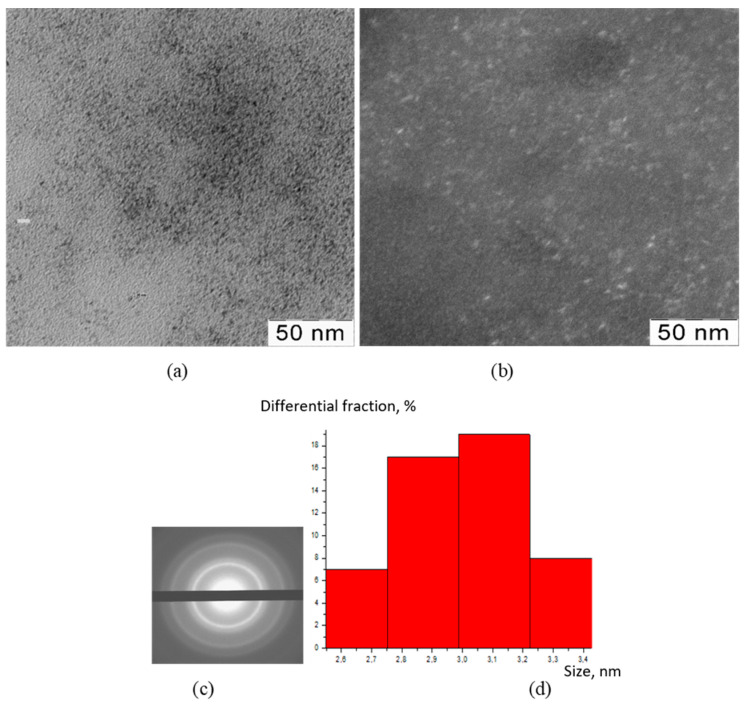
TEM image (**a**), TEM image in dark field (**b**), diffractogram (**c**), nanoparticle size distribution (**d**) of nanoceria-containing composites of CMC with 11.0 wt.% Ce^4+^.

**Figure 4:**
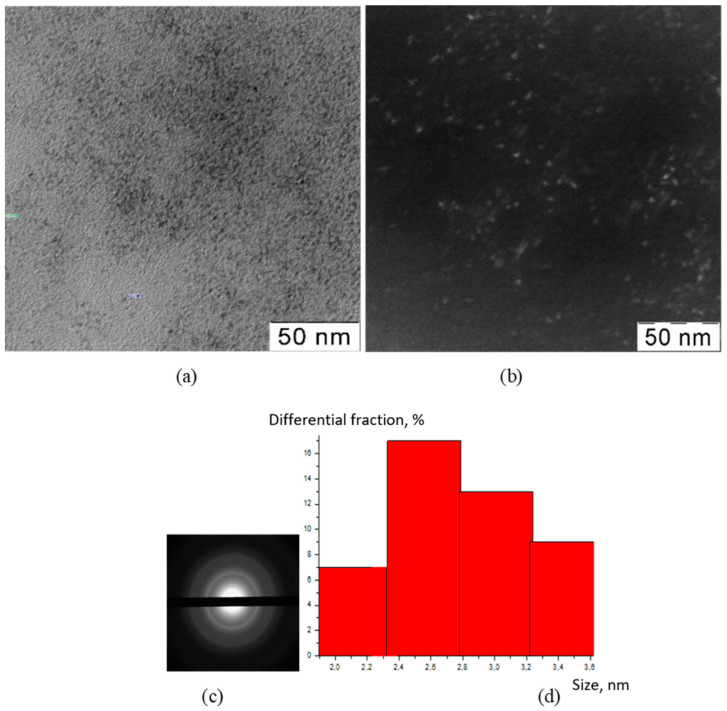
TEM image (**a**), TEM image in dark field (**b**), diffractogram (**c**), nanoparticle size distribution (**d**) of nanoceria-containing composites of CMC with 14.1 wt.% Ce^4+^.

The TEM method was used to visualize the inorganic particles included in the composition of the obtained substances (Figure 1, Figure 2, Figure 3 and Figure 4). The figures present TEM images of samples obtained at various [CMC]/[Ce^4+^] ratios in the reaction mixture.

All presented TEM images demonstrated the presence of dark contrasting nanometer-sized spherical particles. On dark-field TEM images of the samples, nanometer-sized particles appeared as bright spots. This indicates that they have a crystalline structure and represent themselves as sources of diffraction. The presence of a crystalline structure in nanoparticles was confirmed with the electron diffraction patterns, which could be described as a set of diffuse Bragg reflections.

Using TEM images, the sizes of spherical nanoparticles in the composites were calculated. The resulting mean values in all the studied samples were in the range 2–3 nm (for the initial TEM images please see Supplementary Materials Figure S2). Thus, the ratio of components in the reaction mixture did not affect the shape and size of nanoparticles in the obtained products.

**Figure 5:**
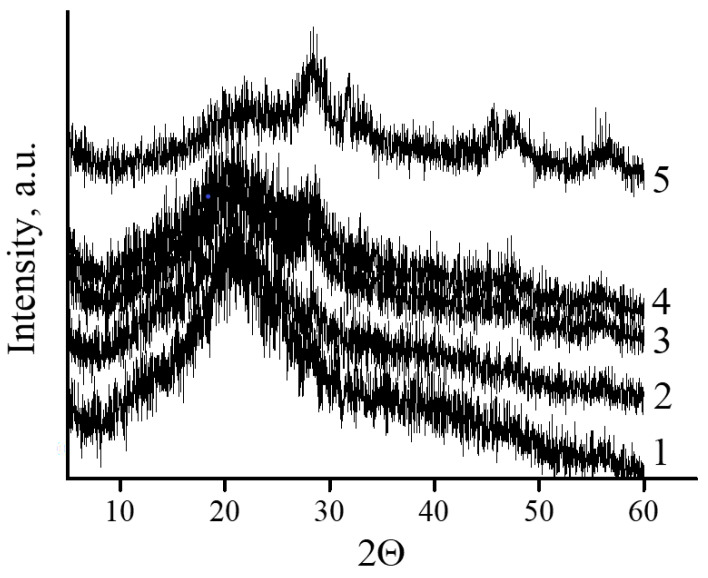
XRD patterns of cerium-containing products based on CMC: pure CMC (**1**); composite with 7.1 wt.% Ce^4+^ (**2**); composite with 9 wt.% Ce^4+^ (**3**); composite with 11.0 wt.% Ce^4+^ (**4**); composite with 14.1 wt.% Ce^4+^ (**5**).

The identification of the crystal structure of the inorganic phase in cerium-containing products based on CMC was carried out using the XRD method (Figure 5).

**Figure 6:**
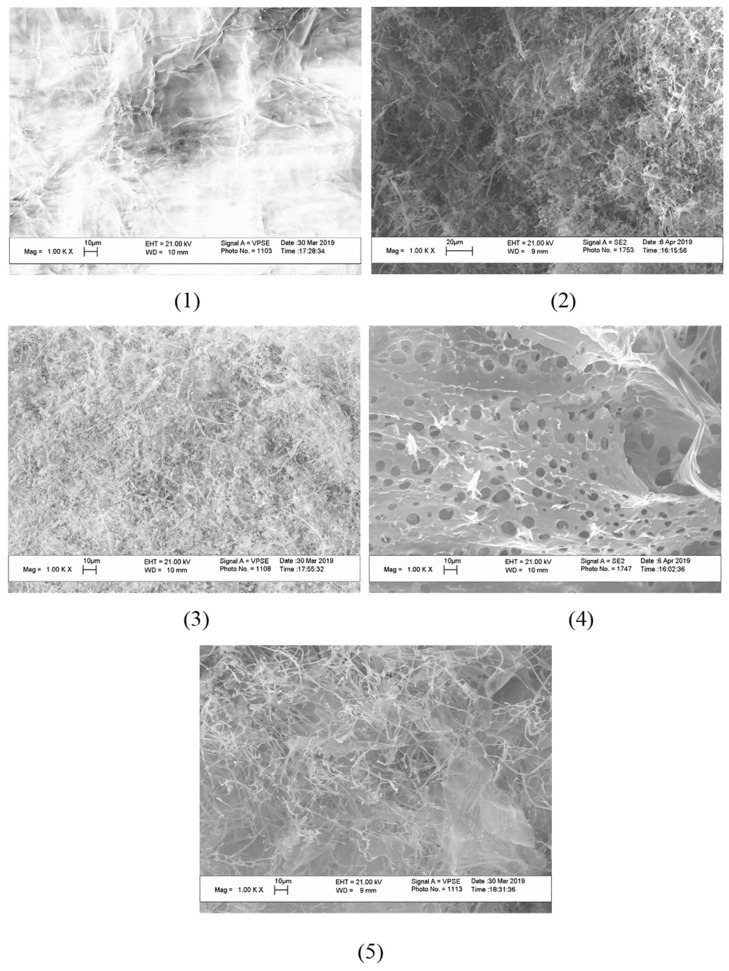
SEM image of cerium-containing products based on CMC: composite with 6.4 wt.% Ce^4+^ (**1**); composite with 7.1 wt.% Ce^4+^ (**2**); composite with 9 wt.% Ce^4+^ (**3**); composite with 11.0 wt.% Ce^4+^ (**4**); composite with 14.1 wt.% Ce^4+^ (**5**).

All diffractograms presented in Figure 6 curves 2–6 had peaks at angles 2θ = 28.4°, 34.38°, 47.6°, and 57°. Broad reflections indicate the presence of a small particle size of the inorganic phase. The diffraction pattern of the original CMC (Figure 6 curve 1) did not contain these peaks.

The microstructure of cerium-containing composites based on CMC was studied using SEM (Figure 6).

An image of the plane surface was obtained with SEM for the initial CMC. The appearance of CeO_2_ nanoparticles in the content of composites resulted in the formation of a porous structure. The pore sizes were practically independent of the mass content of CeO_2_ nanoparticles and varied from 4 to 10 µm.

**Figure 7:**
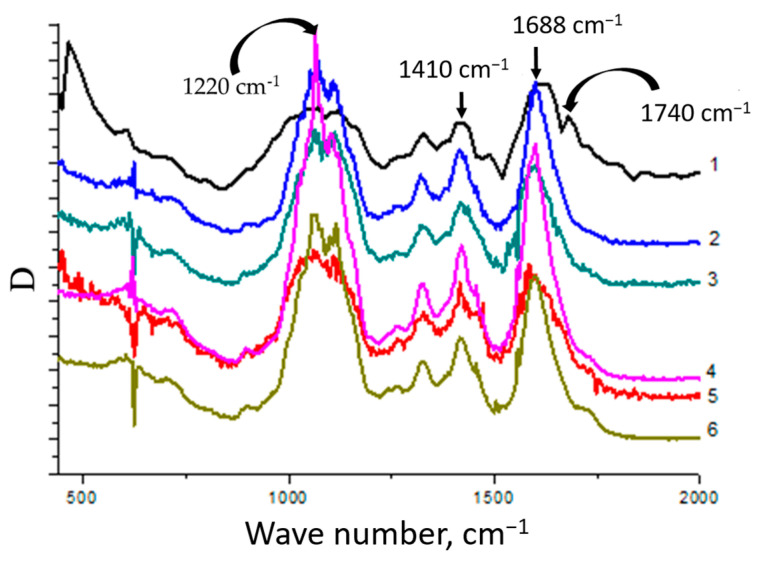
IR spectra of cerium-containing products based on CMC: pure CMC (**1**), composite with 6.4 wt.% Ce^4+^ (**2**); composite with 7.1 wt.% Ce^4+^ (**3**); composite with 9 wt.% Ce^4+^ (**4**); composite with 11.0 wt.% Ce^4+^ (**5**); composite with 14.1 wt.% Ce^4+^ (**6**).

The nature of interactions between CMC macromolecules and cerium oxide nanoparticles was studied using IR spectroscopy (Figure 7).

The CMC spectrum contained bands at wavelengths ν = 1688 cm^−1^ and ν = 1410 cm^−1^, related to antisymmetric and symmetric stretching vibrations of the carboxyl groups of the polyanion, respectively. It should be noted that a wide peak in the range of 1690–1740 cm^−1^, corresponding to the stretching vibrations of the C=O group in the composition of the carboxyl groups of the polyanion, was observed in the spectrum. It is known that the presence of this peak indicates the formation of a system of intra- and intermolecular hydrogen bonds with its participation [34,38]. In addition, in the CMC spectrum there was a band at ν = 1220 cm^−1^, associated with out-of-plane bending vibrations of the hydroxyl groups of the polysaccharide.

In the spectra of the nanocomposites, as the cerium content increased, the absorption intensity decreased in the range of 1690–1740 cm^−1^. This phenomenon is attributed to a violation of the internal structure of the CMC, accompanied by the destruction of the system of hydrogen bonds and the formation of electrostatic contacts between the carboxyl groups of the polysaccharide and the surface of the nanoparticles. In the spectra of the nanocomposites, a shift of the band at ν = 1688 cm^−1^ to the region of lower wave numbers up to ν = 1668 cm^−1^ was observed. The shift of this band is additional evidence that some of the carboxyl groups of the polyanion were involved in an electrostatic interaction with the surface of the cerium oxide nanoparticles. Simultaneously, there is a decrease in the intensity of the band at ν = 1220 cm^−1^. The observed phenomenon indicates that some of the hydroxyl groups of the polyanion did not realize out-of-plane bending vibrations. These hydroxyl groups take part in the interaction with the particles of the inorganic phase, forming coordination bonds with cerium ions on the surface of the nanoparticles.

**Figure 8:**
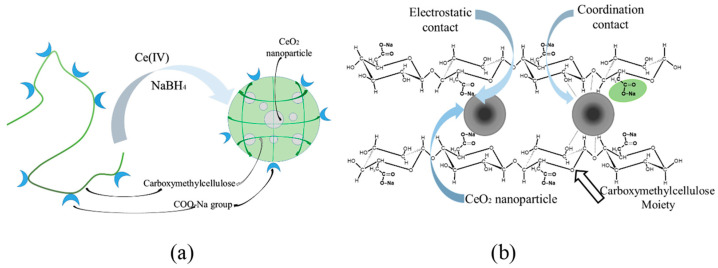
Scheme of formation of the CMC–CeO_2_ composite (**a**); Electrostatic and coordination bond formation between polysaccharide and surface of cerium oxide nanoparticles (**b**).

Thus, nanoparticles are included in the composition of the polymer matrix due to the implementation of interactions between surface cerium ions and functional groups of carboxymethyl cellulose. Carboxyl groups of the polysaccharide form electrostatic contacts with nanoparticles. In addition, the formation of the composite results in the destruction of the system of hydrogen bonds with the participation of carboxyl groups of the initial CMC. Due to this effect, the formation of a system of coordination bonds between the hydroxyl groups of polysaccharides and cerium ions on the surface of nanoparticles is possible. That, in turn, is an additional factor that increases the stability of nanoparticles and their aggregative stability. The scheme of the stabilized nanoparticles is presented in Figure 8.

## 4. Discussion

A one-pot synthesis of fine dispersed CeO_2_ nanoparticles could be carried out at room temperature by direct synthesis in a solution of CMC. The formation of nanoparticles occurs in several stages. First, cerium (IV) from the dissolved (NH_4_)_2_Ce(NO_3_)_6_ undergoes reduction to cerium (III) in the form of cerium hydroxide, which undergoes oxidation by air oxygen dissolved in water. The growth of the nanoparticles is restricted by a stabilizing agent—CMC. Using different CMC-to-cerium molar ratios, a series of nanocomposites was obtained. The morphology and the size of the inorganic nanoparticles stabilized with CMC was studied using TEM. For the all studied compositions of the reaction mixtures the resulting nanoparticles had a spherical shape and a mean diameter of 2–3 nm. Thus, CMC restricts the growth of nanoparticles, providing effective stabilization of the inorganic surface with carboxylate and hydroxyl functional groups. It should be stressed that not only electrostatic but coordination contacts as well provide the stabilization of the nanoparticles. The SEM experiments demonstrated a violation of the continuous film structure of the CMC after the formation of the nanocomposites. In the initial CMC film the intra- and intermolecular hydrogen bonds of the polymer supply the smooth, non-porous structure. The formation of the nanoparticles and their incorporation in the polymer matrix result in destruction of the internal structure of the polymer film due to a change in the function of coordination bonds from stabilization of the film to stabilization of the nanoparticles.

The nature and the structural type of the nanoparticles formed were determined using XRD and TEM analyses. Dark field TEM images of the nanocomposites allows one to estimate the crystalline nature of the inorganic phase. Diffractograms of these particles confirm the crystalline structure of the nanoparticles. However, due to the small size of inorganic particles no detailed information about the structure could be obtained with diffractometer-integrated TEM. The XRD analysis confirmed that the nanoparticles represent themselves as crystals of CeO_2_. For the CeO_2_ powder the characteristic peaks should be observed at angles 2θ = 28.4°, 34.38°, 47.6°, and 57°. The obtained values of 2θ correspond to the following parameters of the CeO_2_ crystalline lattice: 110; 004; 220; 214; 311. With the increase in the inorganic phase share in the nanocomposites, the reflections corresponding to the crystalline lattice of CeO_2_ become more intensive. No additional peaks arise with the increase in the Ce(IV)-to-CMC ratio in the nanocomposite, confirming that the structure of inorganic nanoparticles does not depend on ratio of the components in the reaction mixture.

To support the proposed nature of the stabilization of CeO_2_ nanoparticles with groups of CMC, IR spectroscopy was applied. The initial spectrum of the CMC contains characteristic peaks at 1740 cm^−1^, 1688 cm^−1^, 1420 cm^−1^, and 1220 cm^−1^ reflecting carboxyl, carboxylate, and hydroxyl groups. The formation of nanocomposites results in the vanishing of the 1740 cm^−1^ peak, which reflects the formation of intra- and intermolecular hydrogen bonds. At the same time, the intensive peaks reflecting carboxylate groups were retained in the spectra of the nanocomposites. Hence, the stabilization of the nanoparticles is supplied by electrostatic interactions. Transformation of the 1220 cm^−1^ peak from a wide to a sharp shape with the increase in the cerium content reflects the incorporation of the hydroxyl groups of CMC in interactions with the surface of the inorganic particles.

We suggest that geometrically surrounded nanoparticles of cerium oxide are stabilized with both carboxylate and hydroxyl groups of CMC. The first one ensures electrostatic stabilization while the second one is responsible for the coordination contacts. The dual action of these groups results in effective stabilization of the nanoparticles with a relatively small size.

## 5. Conclusions

Nanocomposites consisting of CeO_2_ nanoparticles stabilized by carboxymethyl cellulose macromolecules were obtained. In the resulting nanocomposites, the content of the inorganic component was determined with UV spectrophotometry. The nanocomposites were also characterized by TEM, SEM, XRD, and IR spectroscopy. It has been found that varying the ratio of the components of the reaction mixture in the course of synthesis makes it possible to obtain nanocomposites with different contents of the inorganic phase while the size and morphology of the nanoparticles did not depend on the reaction mixture composition. The nanoparticles had a spherical shape with an average diameter of 2–3 nm. It was demonstrated that the type of crystal lattice of the nanoparticles corresponds to the structure of CeO_2_.The type of crystal structure did not depend on the ratio of the components in the reaction mixture as well. With the use of a combination of SEM and IR spectroscopy, it has been demonstrated that the stabilization of the nanoparticles is attributed to both electrostatic and coordination contacts of the inorganic surface with the macromolecules. These findings could be useful for the further development and application of cerium oxide nanoparticles.

## Figures and Tables

**Table 1:** Composition of the reaction mixture for the synthesis of cerium-containing nanoparticles and Ce^4+^ yield.

[CMC], Base-Mole/L	(NH_4_)_2_Ce(NO_3_)_6_, mM	[CMC]/[Ce^4+^]	Ce^4+^ in Composite, wt.% *
0.08	0.002	20:1	6.4
0.08	0.0053	15:1	7.1
0.08	0.008	10:1	9.0
0.08	0.011	7:1	11.0
0.08	0.016	5:1	14.1

* Determined with UV spectroscopy.

## Data Availability

Not applicable.

## Supplementary Materials

The following supporting information can be downloaded at: https://www.mdpi.com/article/10.3390/polym15061437/s1, Figure S1: UV spectra of (NH_4_)_2_Ce(NO_3_)_6_ solutions of various concentrations (a): 0.05 mg/mL (1); 0.1 mg/mL (2); 0.15 mg/mL (3); 0.2 mg/mL (4). Calibration plot of absorbance of (NH_4_)_2_Ce(NO_3_)_6_ solutions versus concentration at 310 nm (b).; Figure S2: TEM images of nanoceria-containing composites of CMC with 7.1 wt.% Ce4+ (a); 9.0 wt.% Ce4+ (b); 11.0 wt.% Ce4+ (c); 14.1 wt.% Ce4+ (d).
