# Supplementary material for: One-Step Low Temperature Synthesis of CeO2 Nanoparticles Stabilized by Carboxymethylcellulose
Source: Polymers (Basel). 2023 Mar 14;15(6):1437. doi: 10.3390/polym15061437 (PMC10058267; doi:10.3390/polym15061437)
Supplement: Supplementary file 1 [file polymers-15-01437-s001.zip › polymers-2216899-supplementary.pdf]

Supplementary materials

For

One-Step Low Temperature Synthesis of CeO_2 Nanoparticles Stabilized by Carboxymethylcellulose

Vasily V. Spiridonov [1,*], Andrey V. Sybachin [1], Vladislava A. Pigareva [1], Mikhail I. Afanasov [1], Sharifjon A. Musoev [2], Alexander V. Knotko [2] and Sergey B. Zezin [1]

[1] Department of Chemistry, Lomonosov Moscow State University, Leninskie Gory 1-3, 119991 Moscow, Russia
[2] Faculty of Materials Science, Lomonosov Moscow State University, Leninskie Gory 1-73, 119991 Moscow, Russia

Experimental

UV spectroscopy.

The determination of the cerium content in the composites was carried out using the UV spectroscopy method. The measurements were carried out on a Specord M40 device from Carl Zeiss (Jena, Germany) in the spectral range from 280 to 500 nm. Sample solutions were prepared to record UV spectra. A calibration graph was built according to the method given in [Bumajdad, A.; Eastoe, J.; Mathew, A. Cerium oxide nanoparticles prepared in self-assembled systems. *Adv. Colloid Interface Sci.* **2009**, 147-148, 56–66]. To construct this calibration graph, weighings of cerium ammonium nitrate 0.5 mg, 1 mg, 1.5 mg and 2 mg were dissolved in 100 μl of concentrated H_2SO_4. Then was added 10 ml of an aqueous solution containing 0.1% wt. silver nitrate and 0.2 g ammonium persulfate. After that, the UV spectra of the obtained solutions were recorded in the wavelength range from 200 to 500 nm and the absorption intensity was measured at a wavelength of 310 nm, $D_{\lambda=310\ nm}$. Absorption spectra of solutions containing cerium ions of various concentrations and a calibration plot (dependence against the concentration of cerium ions, mg/ml) are presented in Figure S1.

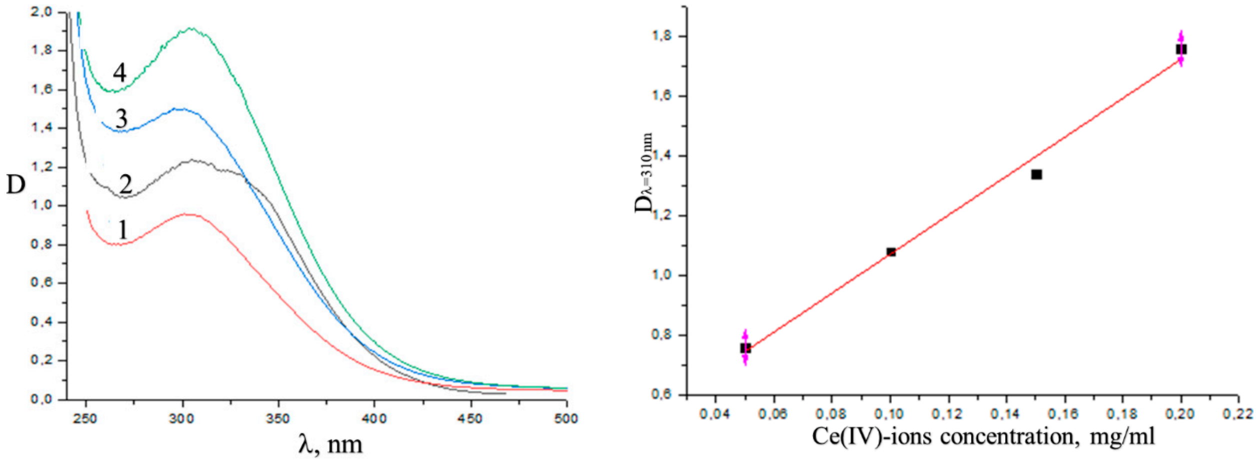

a) b)

Figure S1. UV spectra of $(NH_4)_2Ce(NO_3)_6$ solutions of various concentrations (a): 0.05 mg/ml (1); 0.1 mg/ml (2); 0.15 mg/ml (3); 0.2 mg/ml (4). Calibration plot of absorbance of $(NH_4)_2Ce(NO_3)_6$ solutions versus concentration at 310 nm (b).

TEM imaging.

TEM images of CeO_2/CMC nanocomposites with different content of nanoparticles are presented on Figure S2.

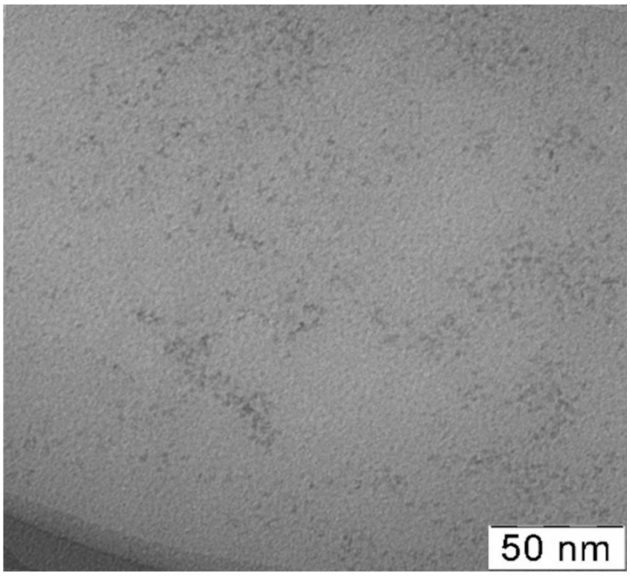

a)

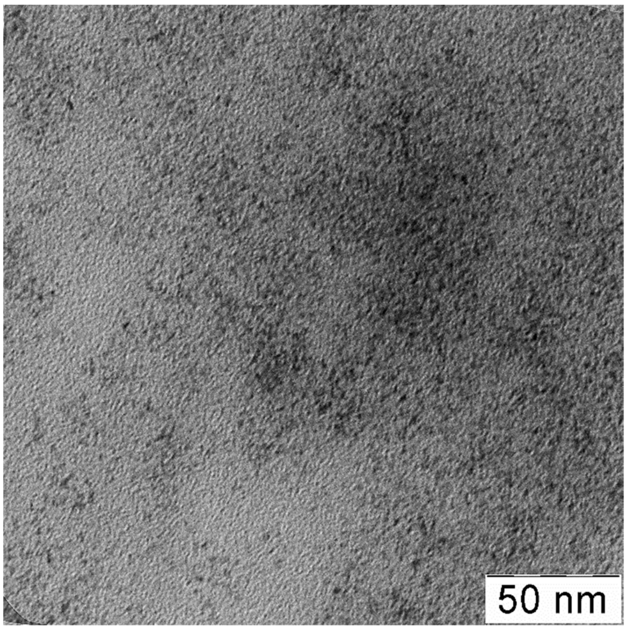

b)

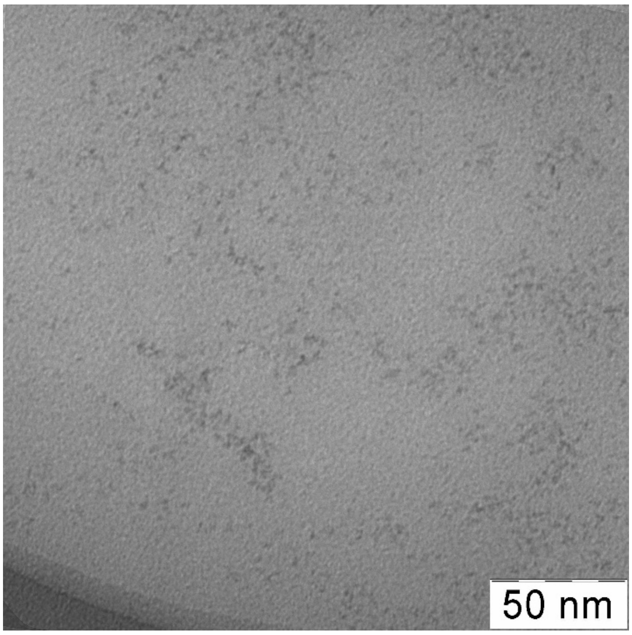

c)

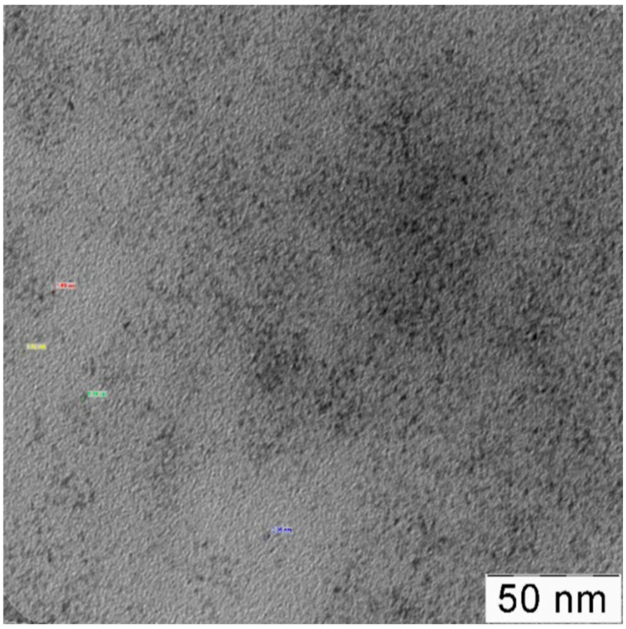

d)

Figure S2. TEM-images of nanoceria-containing composites of CMC with 7.1 wt.% Ce^{4+} (a); 9.0 wt.% Ce^{4+} (b); 11.0 wt.% Ce^{4+} (c); 14.1 wt.% Ce^{4+} (d);
